# Polysomnography Following Traumatic Brain Injury: A Systematic Review and Meta-Analysis

**DOI:** 10.1192/j.eurpsy.2022.2091

**Published:** 2022-09-01

**Authors:** M. Bray, A. Krieg, A. Esagoff, B. Bryant, R. Salas, V. Rao, M. Peters

**Affiliations:** 1 Johns Hopkins University School of Medicine, Department Of Psychiatry And Behavioral Sciences, Baltimore, United States of America; 2 Johns Hopkins University School of Medicine, Department Of Neurology, Baltimore, United States of America

**Keywords:** Polysomnography, sleep disturbances, sleep, traumatic brain injury

## Abstract

**Introduction:**

Sleep disturbances are common following traumatic brain injury (TBI) worsening morbidity and other neuropsychiatric symptoms. Post-TBI alterations in sleep architecture require further study.

**Objectives:**

(1) To evaluate polysomnographic measures of sleep architecture in participants with history of TBI compared to controls and as meta-analyses of pooled means. (2) To evaluate effects of timing and severity of TBI on polysomnographic outcomes.

**Methods:**

PRISMA compliant systematic review was conducted of MEDLINE, PsycINFO, EMBASE and Scopus. Inclusion criteria: 1) reporting polysomnography in the context of TBI and 2) operationalizing TBI using clear/formalized criteria. Data were pooled in random-effects meta-analyses with outcomes expressed as mean differences (MD).

**Results:**

In participants with TBI, sleep was comprised of 19.39% REM sleep, 8.13% N1, 51.18% N2, and 17.53% N3, as determined by meta-analyses of single means. Total sleep time was reduced in chronic (>6 months) TBI compared to acute-intermediate TBI (<6 months) (*p*=0.01). Compared to controls, participants with TBI differed with increased N1 sleep (MD=0.64%; 95%CI=0.02,1.25; *p*=0.04), reduced sleep efficiency (MD=-1.65%; 95%CI=-3.18,-0.12; *p*=0.03), and reduced sleep latency on the multiple sleep latency test (MD=-5.90mins; 95%CI=-10.09,-1.72; *p*<0.01). On sub-group analyses, participants with mild TBI differed from controls with reduced total sleep time (MD=-29.22mins, 95%CI=-54.16,-4.27; *p*=0.02). Similarly, participants with acute-intermediate TBI exhibited increased sleep latency compared to controls (MD=8.96mins; 95%CI=4.07,13.85; *p*<0.01) and differed significantly from participants with chronic TBI (*X^2^* (1,N=608)=6.54; *p*=0.01).

**Conclusions:**

Sleep architecture is altered following TBI with potential implications regarding functional outcomes and recovery. These alterations appear to differ based on severity of injury and time since injury.

**Disclosure:**

No significant relationships.

